# Prevalence, trends, and molecular insights into colistin resistance among gram-negative bacteria in Egypt: a systematic review and meta-analysis

**DOI:** 10.1186/s12941-025-00799-3

**Published:** 2025-05-10

**Authors:** Ahmed Azzam, Haitham Salem, Mahmoud Nazih, Enas Mohamed Lotfy, Fatma E. Hassan, Heba Khaled

**Affiliations:** 1 Department of Microbiology and Immunology, Faculty of Pharmacy, Helwan University, Cairo, Egypt; 2 Faculty of Medicine, Ain Shams University, Cairo, Egypt; 3 Present Address: Al Ryada University for Science and Technology (RST), ElMehwar ElMarkazy-2, Cairo - Alex desert RD K92, 16504 Egypt; 4 Scientific Office, Egyptian Society of Pharmacogenomics and Personalized Medicine (ESPM), Cairo, Egypt; 5 Department of Nursing and Hospital Management, Military Medical Academy, Cairo, Egypt; 6 General Medicine Practice Program, Department of Physiology, Batterjee Medical College, Jeddah, 21442 Saudi Arabia; 7 Medical Physiology Department, Kasr Alainy, Faculty of Medicine, Cairo University, Giza, 11562 Egypt; 8 Department of Biochemistry, Faculty of Pharmacy, Cairo University, Cairo, Egypt; 9 Department of Clinical Pharmacy, Faculty of Pharmacy, Ahram Canadian University, 6th of October City, Giza, 12566 Egypt

**Keywords:** Colistin, Resistance, Gram-negative bacteria, *Mcr*, Egypt, Meta-analysis

## Abstract

**Background:**

This study examines colistin resistance in Gram-negative bacteria in Egypt, analyzing prevalence, trends, geographic variations, colistin-carbapenem resistance correlation, and *mcr*-mediated plasmid resistance.

**Methods:**

We conducted a systematic search of articles published between 2014 and 2024 that reported on colistin or *mcr*-mediated resistance in Gram-negative bacteria isolated from human infections in Egypt, with clearly defined susceptibility testing methods. A random-effects meta-analysis was conducted to estimate colistin resistance prevalence based on broth microdilution (BMD) findings, the gold standard method. To explore the influence of study-level factors—including alternative susceptibility testing methods—a multivariate meta-regression analysis was performed. The results of the meta-regression are reported as regression coefficients (β), representing the difference in colistin resistance, expressed in percentage points. All statistical analyses were conducted using R software.

**Results:**

This analysis included 55 studies. Based on BMD susceptibility testing, colistin resistance was observed in 9% of all recovered Gram-negative isolates (95% CI: 6–14%) and was significantly higher among carbapenem-resistant isolates (31%, 95% CI: 25–38%), with *p* < 0.001. Multivariate meta-regression analysis further confirmed that colistin resistance was significantly higher in carbapenem-resistant isolates compared to the total recovered isolates (β = 9.8% points, *p* = 0.001). Additionally, colistin resistance has significantly increased over time, with a β = 1.8% points per year (*p* = 0.001). The use of the VITEK 2 system was associated with lower detected colistin resistance compared to BMD (β = -7.0, *p* = 0.02). Geographically, resistance rates were higher in Upper Egypt (β = 9.3, *p* = 0.04). Regarding *mcr* plasmid-mediated resistance, *mcr-1* was the most prevalent resistance gene, particularly in *E. coli*. In contrast, *mcr-2* was rare, detected sporadically in *K. pneumoniae* and *P. aeruginosa*.

**Conclusion:**

In Egypt, BMD testing identified colistin resistance in 9% of Gram-negative bacteria, increasing to 31% in carbapenem-resistant isolates. This higher resistance in carbapenem-resistant strains suggests stronger selective pressure from frequent colistin use. Additionally, colistin resistance has shown a rising trend over time, likely driven by increased usage and the spread of plasmid-mediated resistance. These findings underscore the urgent need for strict antimicrobial stewardship and alternative therapies to curb resistance evolution.

**Supplementary Information:**

The online version contains supplementary material available at 10.1186/s12941-025-00799-3.

## Introduction

Gram-negative bacteria are a leading cause of hospital-acquired infections worldwide, posing a significant challenge to healthcare systems [1]. Their outer membrane acts as a barrier against many antibiotics, making infections more difficult to treat. The rapid emergence and spread of multidrug-resistant (MDR) Gram-negative strains have further limited treatment options, leading to higher morbidity and mortality rates [2, 3]. In response, colistin—an antibiotic previously used primarily for topical applications in human medicine due to its nephrotoxic and neurotoxic effects—has re-emerged as a last-resort therapy for MDR infections [4]. However, the increasing reliance on colistin has accelerated the rise of colistin-resistant strains, further complicating infection management [5, 6].

A key mechanism of colistin resistance in Gram-negative bacteria involves modification of the lipopolysaccharide (LPS) layer, which reduces colistin binding and impairs its bactericidal activity [7]. Colistin targets the negatively charged lipid A of LPS, but bacteria can neutralize this charge by adding groups such as phosphoethanolamine (pEtN) or 4-amino-4-deoxy-L-arabinose (L-Ara4N) [4, 8, 9]. Such lipid A modifications are regulated by two-component systems like PmrAB and PhoPQ, with mutations leading to permanent resistance [4]. Additionally, plasmid-borne *mcr* genes, first identified in *E. coli* in 2015 [10], encode enzymes that modify lipid A. This is particularly concerning, as it enables the horizontal transfer of resistance between species—even in the absence of colistin use—posing a significant public health threat [4, 11].

A previous meta-analysis found that colistin resistance in *A. baumannii* had risen significantly, from 2% before 2011 to 5% after 2012 [5]. Similarly, a pooled prevalence analysis of *K. pneumoniae* isolates revealed an increasing trend, with resistance rates rising from 2.89% before 2015 to 2.95% between 2016 and 2019, and a sharp rise to 12.9% in isolates studied from 2020 onward [6].

In Egypt, several meta-analyses have highlighted the alarming burden of antimicrobial resistance [12–14]. Recent evidence indicates widespread use of antimicrobials, including colistin, in animal husbandry across the country. Specifically, colistin was reportedly used in 50% of surveyed farms, with 55% of these applications intended for non-therapeutic purposes such as growth promotion and disease prevention [15]. Despite these concerning trends, there is a notable lack of pooled data on the prevalence of colistin resistance among Gram-negative bacterial isolates, particularly in clinical settings.

To address this gap, we conducted a systematic review and meta-analysis to determine the prevalence of colistin resistance among Gram-negative bacteria isolated from clinically infected patients in Egypt. Our study also aimed to analyze temporal trends, identify geographic variations, assess the correlation between colistin and carbapenem resistance, and explore plasmid-mediated mechanisms underlying colistin resistance. These findings have significant implications for antimicrobial stewardship, infection control strategies, treatment guidelines, and the development of novel therapeutic approaches to combat MDR infections and safeguard colistin’s efficacy.

## Methods

### Search strategy

A comprehensive literature search was conducted to identify studies published between January 1, 2014, and December 5, 2024. The search was carried out using multiple databases, including Web of Science, Google Scholar, Scopus, PubMed, the Egyptian Knowledge Bank, and African Journals Online. Additionally, reference lists of the selected studies were reviewed to ensure thorough coverage of relevant literature.

To ensure systematic organization, a reference library was created to compile the retrieved articles, and duplicate entries were removed using Zotero (version 6). The remaining studies were then screened for eligibility in a stepwise manner—first by title, followed by an abstract evaluation, and finally through a full-text review.

The detailed search strategy, including specific keywords and Boolean operators, is presented in Table S1. Examples of search strategies used in PubMed and Scopus are provided in Table S2. This systematic review was conducted in accordance with the Preferred Reporting Items for Systematic Reviews and Meta-Analyses (PRISMA) guidelines [16], with the PRISMA checklist provided in Table S3.

### Eligibility criteria

The inclusion criteria for this study were as follows: (1) studies of any design reporting data on colistin resistance rates among Gram-negative bacteria or characterizing the plasmid-mediated mcr gene; (2) studies conducted in humans with clinical infections; (3) studies conducted exclusively in Egypt; (4) studies in which the colistin susceptibility method was clearly defined; and (5) studies published between January 1, 2014, and December 5, 2024. This period was selected to capture recent data and reflect the current prevalence in Egypt, ensuring the inclusion of up-to-date research for a comprehensive analysis.

The exclusion criteria were as follows: (1) studies conducted on non-human subjects, such as environmental samples, animals, or food sources; (2) preprints; and (3) studies reporting irrelevant outcomes.

The Clinical and Laboratory Standards Institute (CLSI) and the European Committee on Antimicrobial Susceptibility Testing (EUCAST) recognize broth microdilution (BMD) as the gold standard for detecting colistin resistance [17, 18]. Therefore, we conducted a meta-analysis of colistin resistance prevalence based on this method. However, our study also incorporated other susceptibility testing methods to assess their influence on colistin resistance rates through meta-regression analysis, rather than excluding them.

Two independent authors (H.K. and H.S.) selected relevant articles based on the specified inclusion and exclusion criteria, with cross-checking performed by A.A. and M.N. to ensure accuracy and consistency. Any discrepancies were resolved through discussion and consensus, with input from a third reviewer (F.E.H.) when necessary.

### Data extraction

Data extraction was independently conducted by two reviewers (E.M.L. and F.E.H.) using a standardized Excel sheet, followed by cross-checking by M.N. to ensure accuracy and consistency. Extracted variables included the first author’s last name, publication year, study period, governorate, study setting, colistin susceptibility testing method, total number of isolates tested for colistin susceptibility, number of colistin-resistant isolates, selection criteria for colistin susceptibility testing (total recovered isolates or carbapenem-resistant isolates), tested species, and the presence of *mcr*-mediated plasmid resistance.

### Quality assessment

The quality of the included studies on colistin resistance was evaluated using the Joanna Briggs Institute (JBI) quality assessment tool [19]. This tool assesses the appropriateness of the sample frame and study methods, the adequacy of the sample size, and the clarity in describing study subjects and settings. It also evaluates the validity of colistin resistance detection methods, the reliability of susceptibility testing, and the appropriateness of statistical analyses. Two independent reviewers (E.M.L. and F.E.H.) conducted the assessments, with discrepancies resolved by A.A. The checklist items from the JBI Critical Appraisal Tool are provided in Table S4.

### Statistical analysis

A meta-analysis was conducted to estimate colistin resistance prevalence using a random-effects model with inverse-variance weighting. The pooled colistin resistance rate and 95% confidence interval (CI) were reported based on the BMD method, the gold standard for colistin susceptibility testing. A sensitivity analysis was conducted using the leave-one-out method to assess the stability of the findings. A multivariate meta-regression analysis was conducted to explore potential sources of heterogeneity, including isolate selection criteria for colistin susceptibility testing (e.g., carbapenem-resistant, MDR, XDR, or all recovered isolates), geographic region, susceptibility testing method, and study period. The study period was treated as a continuous variable to assess potential time trends. For studies conducted over two or more years, the midpoint of the study period was calculated and used in the analysis. Categorical variables were treated as factors, and only moderators with at least five estimates were included to ensure statistical robustness. A restricted maximum likelihood regression model was applied to evaluate the influence of these variables on colistin resistance rates, with results reported as regression coefficients (β) and 95% confidence intervals (CIs). Here, β represents the difference or change in colistin resistance, expressed in percentage points.

For categorical variables, it reflects the difference in resistance between each category and the reference group. For continuous variables, such as study year, it indicates the change in resistance per year. Statistical analyses were performed using R software (version 4.4.1), and a *p*-value < 0.05 was considered statistically significant.

## Results

### Characteristics of the included studies

**Fig. 1:**
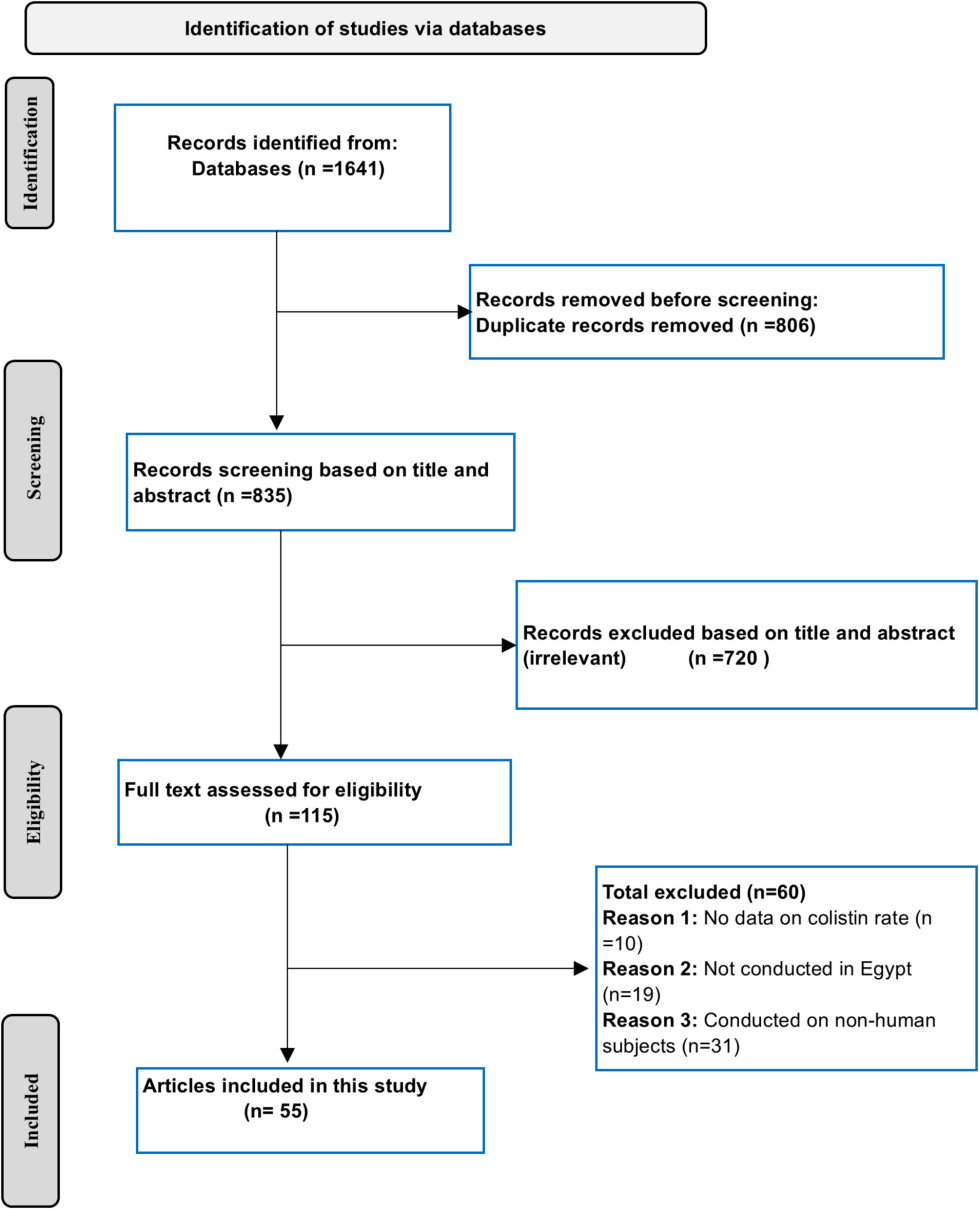
The PRISMA flow chart depicting the studies selection process

A total of 1,641 studies were reviewed. Of these, 55 studies were included in this meta-analysis, with publication years ranging from 2014 to 2024 and study periods spanning from 2012 to 2022 [20–74], as shown in Table 1. The selection process of the included studies is visualized in Fig. 1.

**Table 1:** Characteristics of the included studies

Last name of first author	Publication year	Study period	Governorate	Setting	Susceptibility test for colistin	Total tested for colistin	No. of colistin-resistant isolates	Isolates chosen for Colistin susceptibility testing	Tested Species	Quality score (Out of 8)
Shawky [68]	2015	NA	Alexandria	Alexandria Main University Hospital	E-test	65	9	CR	*K. pneumoniae*	5
Azzab [67]	2016	2014–2015	Zagazig	Zagazig University Hospital	DD	37	0	Total	*Klebsiella Spp.*	6
Alkasaby [66]	2017	2014–2016	Mansoura	Mansoura University Hospital	E-test	280	9	Total	*A. baumannii*	7
Assem [64]	2017	2013	Cairo	Cairo University Hospital	DD	50	0	Total	*A. baumannii*, *K. pneumoniae*, *P. aeruginosa*, *and E. coli​.*	6
Ghonaim [63]	2017	2014–2016	Zagazig	Zagazig University Hospitals	VITEK-2	22	0	ESBL	*E. coli*, *K. pneumoniae*, *E. cloacae*	*6*
Abdulall [62]	2018	2015–2016	Cairo	ICU (3 tertiary hospital units)	DD	57	0	Total	*E. cloacae*, *E. coli*, *A. baumannii*, *and P. aeruginosa​*	*6*
Abdulzahra [61]	2018	Jan– Jul 2015	Cairo	El-Kasr El-Ainy Hospital	BMD	40	2	CRE	*A. baumannii*	*7*
El-Mahallawy [76]	2018	2015	Cairo	National Cancer Institute	VITEK-2	96	0	Total	*K. pneumoniae*, *E. coli*	*6*
El-Masry [60]	2018	2016–2018	Giza	Giza Chest Hospital	VITEK-2	22	1	Total	*A. baumannii*	*6*
Awad [59]	2019	2017–2018	Menoufia	National Liver Institute	VITEK-2 not	38	0	Total	*K. pneumoniae*, *E. coli*, *M. morganii.*	*6*
Emara [58]	2019	2017–2018	Tanta	Tanta University Hospitals	BMD	61	10	Total	*K. pneumoniae*, *E. coli*, *P. aeruginosa*	*7*
Mokhtar [57]	2019	2017–2018	Assiut, Minia	Assiut & Minia University Hospitals	BMD	100	22	MDR	*E. coli*	*8*
Sokkary [56]	2019	June 2017– May 2018	Zagazig	Zagazig University Hospitals	BMD	1,218	34	MDR	*E. coli*, *K. pneumoniae.*	*8*
Zafer [55]	2019	Jan 2016– June 2017	Cairo	National Cancer Institute	BMD	450	40	Total	*K. pneumoniae*, *E. coli*	*8*
Basha [53]	2020	2017	Giza	Kasr El Aini Hospital & El Borg Laboratory	BMD	100	0	Total	*P. aeruginosa*	*8*
El-Baky [72]	2020	2016–2017	Minia	Minia University Hospital	AD	72	16	MDR	*P. aeruginosa*	*6*
Fam [52]	2020	2015–2016	Giza	Theodor Bilharz Research Institute	BMD	17	9	CR	*A. baumannii*	*7*
Rabie [51]	2020	Jan - Aug 2019	Zagazig	Zagazig University Hospitals	BMD	200	24	Total	*E. coli*, *K. pneumoniae*	*8*
Raheel [50]	2020	Nov 2016 - Dec 2018	Ismailia	Suez Canal University Hospitals	BMD	53	21	CR	*E. coli*, *K. pneumoniae*	*7*
Shabban [49]	2020	June - Dec 2019	Cairo	Ain Shams University Hospital	E-test	60	4	MDR	*K. pneumoniae*, *P. aeruginosa*, *A. baumannii*, *E. coli*	*6*
El-Mokhtar [48]	2021	2017–2019	Assiut	Assiut University Hospital	BMD	140	21	Total	*E. coli*	*8*
Elshimy [47]	2021	2018–2019	Cairo	Multiple Hospitals in Cairo	E-test	470	3	Total	*E. coli*	*7*
Ibrahim [46]	2021	2019	Cairo	Ain Shams University Hospitals	BMD	100	14	Total	*K. pneumoniae*, *P. aeruginosa*, *E. coli*, *Citrobacter*	*8*
Khattab [71]	2021	2020–2021	Ismailia	Suez Canal University Hospitals	BMD	116	43	CR	*E. coli*, *K. pneumoniae*	*8*
Mashalya [77]	2021	2016–2019	Mansoura	Mansoura University Hospitals	BMD	115	7	Total	*E. cloacae*, *E. aerogenes*	*8*
Negm [45]	2021	January 1, 2019– December 31, 2019	Zagazig	Zagazig University Hospitals	VITEK-2	31,638	2,141	Total	*K. pneumoniae*, *E. coli*, *P. aeruginosa*, *A. baumannii*	*7*
Ajlan [44]	2022	Nov 2020– Mar 2022	Menoufia	Menoufia University Hospitals	BMD	155	43	CR	*E. coli*, *K. pneumoniae*, *P. aeruginosa*, *A. baumannii*, *Enterobacter spp.*, *Citrobacter spp.*	*8*
Badran [42]	2022	2021	Zagazig	Zagazig University Hospitals	CBDE	90	20	CR	*K. pneumoniae*, *E. coli*, *P. mirabilis*	*6*
Defrawy [41]	2022	2016–2017	Giza	Theodor Bilharz Research Institute	VITEK-2	50	0	CR	*K. pneumoniae*, *E. coli*, *E. cloacae*, *S. marcescens*, *C. freundii*	*6*
El-Din [40]	2022	2020–2022	Sohag	Sohag University Hospitals	E-test	75	18	Total	*P. aeruginosa*	*6*
Gaballah [39]	2022	2018–2019	Alexandria & El Behira	El Shatby University Hospital, Mabart Al Asafra Hospital, Damanhur General Hospital	VITEK-2	336	18	Total	*K. pneumoniae*, *E. coli*, *A. baumannii*, *P. aeruginosa*, *S. maltophilia*	*7*
El-Mahallawy [65]	2022	2019	Cairo	National Cancer Institute	BMD	196	39	MDR	*K. pneumoniae*, *E. coli*, *E. cloacae*	*8*
Mohamed [36]	2022	Not specified	Cairo	Ain Shams University Hospitals	VITEK-2	25	3	Total	*P. aeruginosa*	*5*
Mostafa [43]	2022	2018–2019	Cairo	Abbassia Fever Hospital	E-test	200	9	Total	*E. coli*, *K. pneumoniae*, *A. baumannii*, *P. aeruginosa*, *Salmonella spp.*	*7*
Ramadan [35]	2022	Jan 2020 - Nov 2021	Zagazig	Zagazig University Hospitals	CBDE	73	8	Total	*K. pneumoniae*	*6*
Shrief [34]	2022	Sep 2021– Mar 2022	Mansoura	Mansoura University Hospitals	BMD	92	30	CR	*E. coli*, *K. pneumoniae*	*7*
Sorour [33]	2022	April– November 2019	Cairo	Cairo University Hospitals	BMD	115	12	Total	*E. coli*, *K. pneumoniae*, *P. aeruginosa*, *A. baumannii*	*8*
Abdelbary [31]	2023	Sep 2022– Feb 2023	Assiut	Assiut University Children’s Hospital	BMD	56	15	Total	*K. pneumoniae*	*7*
Abozahra [30]	2023	Jan– Jul 2022	Beheira	Damanhour Medical National Institute	BMD	82	32	Total	*K. pneumoniae*	*7*
Elshamy [29]	2023	2020–2021	Cairo	Kasr Al-Ainy & El-Demerdash Tertiary Care Hospitals	BMD	19	1	CR	*E. coli*, *K. pneumoniae*, *P. aeruginosa*, *A. baumannii*	*7*
Khatib [28]	2023	2020–2022	Cairo	Multiple Hospitals	BMD	270	11	Total	*K. pneumoniae*, *P. aeruginosa*	*8*
Mahmoud [27]	2023	2021–2022	Cairo	Ain Shams University ICU	BMD	84	36	MDR	*K. pneumoniae*, *E. coli*	7
Mohamed [37]	2023	2017–2020	Alexandria	ICU in a tertiary care hospital	VITEK-2	443	5	Total	*K. pneumoniae*, *E. coli*, *P. aeruginosa*, *A. baumannii*	*7*
Abdel-Aty [26]	2024	Aug 2022– Feb 2023	Cairo	Kasr Al-Ainy University Hospitals	BMD	250	57	Total	*E. coli*, *K. pneumoniae*, *P. aeruginosa*, *A. baumannii*	*8*
Afify [25]	2024	Aug 2020– Apr 2021	Alexandria	Mabaret El Asafra Laboratories	BMD	111	11	Total	*K. pneumoniae*, *P. aeruginosa*, *A. baumannii*	*8*
Ali [24]	2024	Mar 2019– Feb 2022	Zagazig	Zagazig University Hospitals	VITEK-2	57	8	CR	*K. pneumoniae*	*6*
Alshaikh [23]	2024	2021–2022	Tanta	Tanta University Hospital	BMD	100	0	Total	*E. coli*	*8*
Edward [22]	2024	2017–2018	Alexandria	Alexandria Main University Hospital	BMD	104	6	Total	*P. aeruginosa*	*8*
El-Kholy [74]	2024	2021	Alexandria	Mabaret El Asafra Laboratories	BMD	94	27	CR	*K. pneumoniae*	*7*
Makled [73]	2024	2021–2023	Menoufia	Menoufia University Hospitals	AD	80	10	Total	*P. aeruginosa*, *A. baumannii*	*6*
Mohamed [38]	2024	2022	Zagazig	Zagazig University Hospitals	VITEK-2	26	3	CR	*K. pneumoniae*	*6*
Elnahriry [21]	2016	2015	Cairo	Hospital (ICU patient)	BMD	241	1	Total	*E. coli*, *K. pneumoniae*, *P. aeruginosa*, *A. baumannii*	*8*
Attalla, [70]	2023	July - December 2020	Alexandria	Private hospital laboratory with 8 satellite branches covering Alexandria	BMD	NA	17	NA	*K. pneumoniae*	*8*
Osama [20]	2019	2016 − 2017	Cairo	Two teaching hospitals and two private clinical labs	DD	30	5	CR	*E. coli*, *K. pneumoniae*, *P. aeruginosa*,	*6*
Al-Agamy [69]	2014	Jan - Mar 2012	Cairo	Kasr El Aini Hospital, Dar Al Fouad Hospital	AD	40	2	CR	*A. baumannii*	*6*

BMD (Broth Microdilution), DD (Disk Diffusion), AD (Agar Dilution), CBDE (Colistin Broth Disk Elution), CRI (Carbapenem-Resistant Isolates), MDR (Multidrug-Resistant Isolates), ESBL (Extended-Spectrum Beta-Lactamase-Producing Isolates), NA (Not available)

The included studies employed various susceptibility testing methods for colistin. The BMD method was the most commonly used, appearing in 28 studies. The VITEK 2 automated system was utilized in 11 studies, while the E-test was used in 6 studies. Other methods included disk diffusion (DD) in 4 studies, agar dilution (AD) in 3 studies, and colistin broth disk elution (CBDE) in 2 studies. All included studies used standardized breakpoints based on either CLSI or EUCAST guidelines.

The quality of the included studies, as evaluated using the JBI Critical Appraisal Tool, indicates that all studies achieved a minimum score of five out of eight, which we considered the threshold for fair quality, as presented in Table 1. Lower scores in some studies were primarily attributed to the lack of study setting details, the absence of a defined study period, the use of methods other than the BMD method, and small sample sizes. The detailed quality scores of the included studies are presented in Table S5.

### Prevalence of colistin resistance among Gram-Negative Bacteria in Egypt based on broth microdilution method

**Fig. 2:**
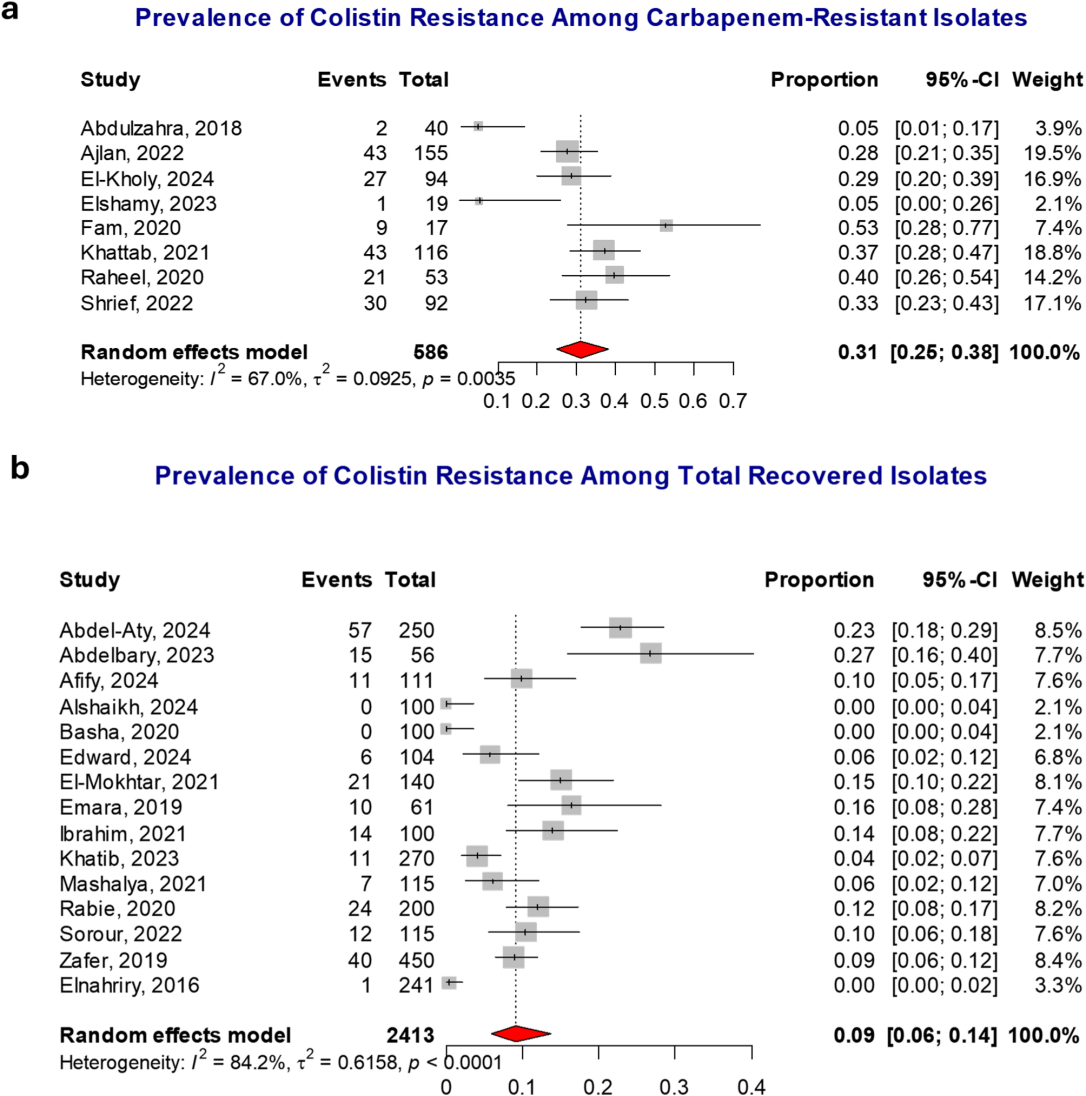
Pooled colistin resistance rate among Gram-negative bacteria isolated from patients with infections in Egypt, based on the random-effects model. (**a**) Colistin resistance rate among carbapenem-resistant isolates: 31% (95% CI: 25–38%). (**b**) Colistin resistance rate among all recovered isolates: 9% (95% CI: 6–14%)

Colistin resistance was observed in 9% of all recovered isolates (95% CI: 6–14%, I² = 84.2%). Among carbapenem-resistant isolates, the prevalence was significantly higher at 31% (95% CI: 25–38%, I² = 67%). This difference is statistically significant, as indicated by a *P* value of < 0.001, as shown in Fig. 2.

**Fig. 3:**
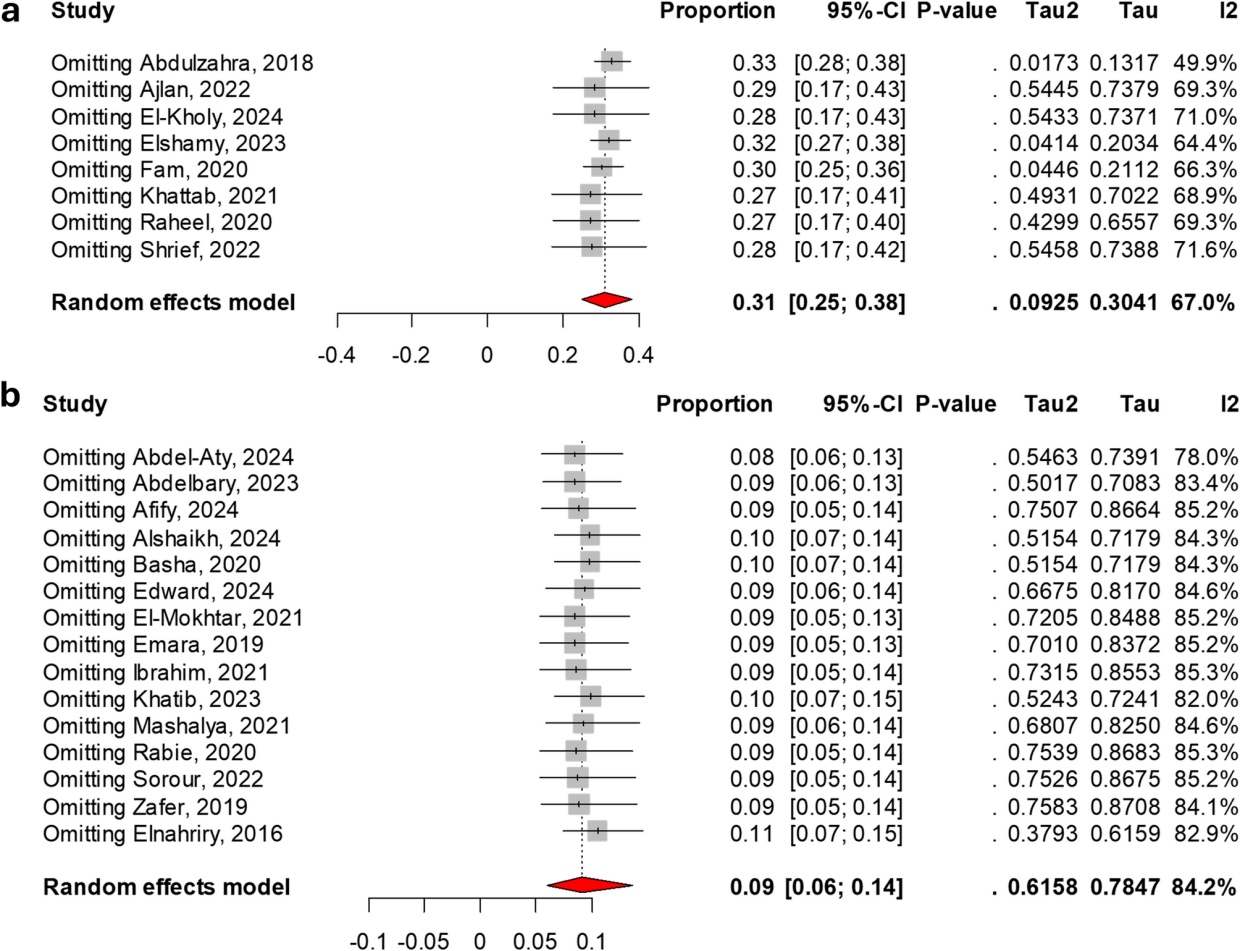
Leave-One-Out Sensitivity Analysis of Colistin Resistance Rates Based on the Random-Effects Model. (**a**) The colistin resistance rate among carbapenem-resistant isolates varied by up to 4% following the exclusion of specific studies. (**b**) The colistin resistance rate among total recovered isolates exhibited a maximum shift of 2% upon the removal of any single study

Using a leave-one-out sensitivity analysis, we found that colistin resistance among all recovered isolates showed minimal variation, with prevalence shifting by no more than 2% upon excluding any single study. In contrast, colistin resistance among carbapenem-resistant isolates fluctuated by up to 4% following the removal of certain studies [50, 71], as shown in Fig. 3.

### Multivariate Meta-Regression analysis of colistin resistance among Gram-Negative Bacteria in Egypt

Multivariate Meta-Regression Analysis of Colistin Resistance Among Gram-Negative Bacteria in Egypt.

The results of the multivariate meta-regression analysis are summarized in Table 2. To ensure statistical robustness, only moderators with at least five estimates were included. A total of 46 studies were analyzed, with an 𝑅² of 53%, indicating that these moderators collectively explain more than half of the between-study variation. The analysis showed that colistin resistance was 9.8% points higher in carbapenem-resistant isolates compared to all recovered isolates (95% CI: 3.9–15.7, *p* = 0.001). Additionally, colistin resistance increased by 1.8% points per year over the study period (95% CI: 0.7–2.8, *p* = 0.001), indicating a significant upward trend. In contrast, the VITEK 2 automated system reported colistin resistance rates that were 7% points lower than those obtained using BMD method (95% CI: -13 to -1, *p* = 0.02). A similar trend was observed when comparing E-test and disc diffusion methods to BMD, with 3% points lower resistance, but this difference was not statistically significant (95% CI: -9.2 to 3.2, *p* = 0.35). Regarding geographic variation, colistin resistance was 9.3% points higher in Upper Egypt compared to Greater Cairo (95% CI: 0.5–18, *p* = 0.04). No significant difference was found for the Delta Region (difference of 0.5% points; 95% CI: -4.4 to 5.4, *p* = 0.83).

**Table 2:** Multivariate Meta-Regression analysis of colistin resistance among Gram-Negative Bacteria in Egypt

Moderators	Regression coefficient (β)¥	CI Lower	CI Upper	*P*-value	Significance
**Preselected resistant phenotypes for colistin susceptibility testing (Reference = total recovered isolates)**					
Carbapenem resistance	9.8	3.9	15.7	0.001	**
Multi-drug resistance	5.7	− 1.9	13.4	0.14	
**Region (Ref: Greater Cairo)**					
Delta Region	0.5	− 4.4	5.4	0.83	
Upper Egypt	9.3	0.5	1.8	0.04	.*
**Susceptibility method**, **Reference = Broth Microdilution**					
Agar diffusion-based susceptibility testing methods (E-test and Disc Diffusion Methods)	− 3.0	-9.2	3.2	0.35	
VITEK 2 system	-7.0	-13	-1.0	0.02	*
**Study Period: 2012–2022**					
Year	1.8	0.7	2.8	0.001	**

* indicates *P* value < 0.05 to 0.01; ** indicates *P* value < 0.01 to 0.001

¥ The β coefficient is expressed in percentage points

### Prevalence of *mcr*-Mediated plasmid resistance among Colistin-Resistant isolates

Twenty studies provided data on the prevalence of *mcr*-mediated plasmid resistance among colistin-resistant isolates, as shown in Table 3. Overall, *mcr-1* remains the most commonly identified resistance gene in clinically relevant Gram-negative bacteria, particularly *E. coli.*

**Table 3:** Distribution of *Mcr* variants among Colistin-Resistant Gram-Negative bacteria

Bacteria Species (mcr Variant)	Author, Publication year	No. of Colistin-Resistant Isolates	No. of Resistant Isolates Harboring mcr genes (%)
***E. coli*** (***mcr-1***)	EL-Mokhtar, 2019 [57]	22	22 (100%)
	El-Mokhtar, 2021 [48]	21	21 (100%)
	Elshimy, 2021 [47]	3	3 (100%)
	Elnahriry, 2016 [21]	1	1 (100%)
	Mahmoud, [27]	6	6 (100%)
	Rabie, 2020 [51]	8	1 (12.5%)
	Ajlan, 2022 [44]	13	3 (23.1%)
	El-Sokkary, 2019 [56]	19	1 (5.3%)
	Zafer, 2019 [55]	18	1 (5.5%)
	Emara, 2019 [58]	1	0 (0.0%)
	Ibrahim, 2021 [46]	1	0 (0%)
***E. coli*** (***mcr-2***)	El-Mokhtar, 2021 [48]	22	0 (0.0%)
	Rabie, 2020 [51]	8	0 (0.0%)
	El-Sokkary, 2019 [56]	19	0 (0.0%)
	Zafer, 2019 [55]	18	0 (0.0%)
	Mahmoud, 2023 [27]	6	0 (0%)
***K. pneumoniae*** (***mcr-1***)	Abdelbary, 2023 [31]	15	15 (100%)
	Abozahra, 2023 [30]	32	27 (84.4%)
	Mahmoud, 2023 [27]	30	28 (93.3%)
	Rabie, 2020 [51]	16	1 (6.3%)
	Ibrahim, 2021 [46]	10	1 (10%)
	Zafer, 2019 [55]	22	1 (4.5%)
	Attalla, 2023 [70]	17	1 (5.8%)
	Emara, 2019 [58]	8	0 (0%)
	Afify, 2024 [25]	11	0 (0%)
	Khatib, 2023 [28]	8	0 (0%)
	El-Sokkary, 2019 [56]	15	0 (0.0%)
	Ajlan, 2022 [44]	17	0 (0%)
***K. pneumoniae*** (***mcr-2***)	Mahmoud, 2023 [27]	30	10 (33.3%)
	Afify, 2024 [25]	11	0 (0%)
	Abdelbary, 2023 [31]	15	0 (0%)
	Rabie, 2020 [51]	16	0 (0%)
	El-Sokkary, 2019 [56]	15	0 (0%)
	Zafer, 2019 [55]	22	0 (0%)
	Ajlan, 2022 [44]	17	0 (0%)
***P. aeruginosa*** (***mcr-1***)	El-Din, 2022 [40]	18	10 (55.6%)
	Shabban, 2020 [49]	2	1 (50%)
	El-Baky, 2020 [72]	16	8 (50%)
	Emara, 2019 [58]	1	0 (0%)
	Khatib, 2023 [28]	3	0 (0%)
	Ibrahim, 2021 [46]	3	0 (0%)
***P. aeruginosa*** (***mcr-2***)	El-Din, 2022 [40]	18	8 (44.4%)
	Khatib, 2023 [28]	3	1 (33.3%)
	El-Baky, 2020 [72]	16	0 (0%)
***A. baumannii*** (***mcr-1***)	Shabban, 2020 [49]	2	2 (100%)
	Ajlan, 2022 [44]	4	0 (0%)
***A. baumannii*** (***mcr-2***)	Ajlan, 2022 [44]	4	0 (0%)

Among *E. coli* isolates, 45.5% (5/11) of studies reported a 100% prevalence of *mcr-1*, while four studies observed prevalence rates ranging from 5.5 to 23%. Only two studies, which tested a single isolate, did not detect the gene. For *K. pneumoniae*, 8.3% (1/12) of studies reported a 100% prevalence of *mcr-1*, six studies documented prevalence rates between 4.5% and 93.3%, and five studies found no evidence of the gene (0%). In *P. aeruginosa*, 50% (3/6) of studies reported an *mcr-1* prevalence of 50% or more among the isolates, while the remaining three studies did not detect the gene. Data on *A. baumannii* are limited, with only two studies available, one reporting a 100% prevalence in its sample and the other reporting 0%.

By contrast, *mcr-2* appears to be less prevalent overall. Although it has been identified in some studies involving *K. pneumoniae and P. aeruginosa*, no detection was reported in any of the five *E. coli* studies. Among seven studies on *K. pneumoniae*, only one (out of 7) found a 33.3% prevalence of *mcr-2*, whereas the remaining six reported no detection. For *P. aeruginosa*, two of three studies observed detection rates of 44.4% and 33.3%, respectively, while the third did not detect *mcr-2*. Only one study on the presence of *mcr-2* in *A. baumannii* reported 0% prevalence.

## Discussion

Colistin is widely regarded as a last-resort antibiotic for treating infections caused by MDR Gram-negative bacteria, particularly carbapenem-resistant strains [75]. Its increasing use in clinical settings—especially where alternative treatment options are limited—has raised significant concerns about the emergence and spread of resistance. In this context, effective surveillance is critical for the early detection of resistance patterns, monitoring geographic and temporal trends, guiding empirical therapy, informing infection control strategies, supporting antimicrobial stewardship efforts, and shaping public health policies.

In light of these concerns, this meta-analysis revealed that 9% of all Gram-negative isolates were resistant to colistin, with the rate increasing to 31% among carbapenem-resistant strains. This sharp rise underscores the growing selective pressure associated with colistin use in the treatment of carbapenem-resistant infections. Furthermore, the significant upward trend in resistance over time suggests that, as colistin usage becomes more common, resistant phenotypes are more readily selected. This pattern is further complicated by regional variability, with Upper Egypt exhibiting higher resistance rates compared to other areas, potentially reflecting distinct antibiotic usage practices or healthcare infrastructures. Equally noteworthy is the distribution of plasmid-mediated resistance, where the *mcr-1* gene predominated across multiple species—especially in *E. coli*—while *mcr-2* emerged only sporadically. These collective observations underscore the urgent need to revisit treatment protocols, implement robust antimicrobial stewardship programs, and develop novel therapeutic strategies to stem the further spread of colistin resistance.

Our analysis estimated a pooled colistin resistance prevalence of 9% among all recovered Gram-negative isolates (95% CI: 6–14%), with a significantly higher resistance rate of 31% among carbapenem-resistant isolates (95% CI: 25–38%, *p* < 0.001). These findings were derived using the BMD method, which is considered the gold standard for colistin susceptibility testing. Moreover, our multivariate meta-regression analysis confirmed that colistin resistance was significantly higher in carbapenem-resistant isolates than in all recovered isolates (β = 9.8, 95% CI: 3.9–15.7, *p* = 0.001). Our findings are notably higher than global estimates reported in previous meta-analyses, which found colistin resistance rates of 3.1% (95% CI: 1.5–4.7%) for *K. pneumoniae* and 4% (95% CI: 3–5%) for *A. baumannii* [5, 6]. A meta-analysis conducted in Iran reported a colistin resistance rate of 31.7% (95% CI: 12.4–60.2%) among carbapenemase-producing *K. pneumoniae*, which was significantly higher than the 6.9% (95% CI: 3.6–12.8%) observed across all isolates [77]. This higher colistin resistance rate among carbapenem-resistant isolates compared to all recovered isolates can be explained by two key mechanisms: selective pressure from colistin use and genetic co-transfer of resistance mechanisms. Since carbapenem-resistant infections often leave colistin as one of the last available treatment options, its frequent use exerts strong selective pressure, favoring the survival and proliferation of bacterial subpopulations that possess or acquire colistin resistance. Additionally, carbapenem resistance is frequently mediated by carbapenemase genes (e.g., *KPC*, *NDM*, *OXA-48*), which are commonly located on mobile genetic elements such as plasmids and transposons. These elements may also carry colistin resistance genes (e.g., *mcr-1* to *mcr-10*), facilitating the co-transfer of resistance traits. Even in the absence of *mcr* genes, plasmids often harbor other resistance determinants, promoting MDR profiles that further contribute to colistin resistance. The clinical significance of the high colistin resistance observed in carbapenem-resistant isolates is profound, as it renders one of the few remaining therapeutic options ineffective, leading to increased mortality, morbidity, and the risk of untreatable infections.

Additionally, colistin resistance has significantly increased over time, with a β = 1.8% points per year (*p* = 0.001). This finding is consistent with previous meta-analyses, which demonstrated a similar upward trend through subgroup analyses based on study periods [5, 6, 77]. However, we employed multivariate analysis instead of subgroup analysis to generate more robust and reliable estimates, ensuring that the observed increase in colistin resistance reflects a true temporal trend rather than being influenced by regional variations, differences in the type of isolates selected for susceptibility testing (e.g., CR, MDR, or XDR), or methodological discrepancies in susceptibility testing across studies.

Additionally, geographic disparities were evident. Colistin resistance rates were significantly higher in Upper Egypt compared to Greater Cairo (β = 9.3% points, *p* = 0.04). This disparity may be attributed to several factors associated with Upper Egypt—the southern region of the country—including limited access to healthcare services, deeply rooted traditional practices, higher illiteracy rates, and greater levels of poverty compared to Greater Cairo and the Delta region [78]. These findings highlight the urgent need for targeted resource allocation, including improved surveillance, antimicrobial stewardship programs, and public health interventions, to mitigate resistance in underserved regions.

In contrast, the evaluation of susceptibility testing methods revealed that the VITEK 2 automated system was associated with lower colistin resistance rates compared to the BMD method (β = -7.0, 95% CI: -13.0 to -1.0, *p* = 0.02). A similar trend was observed when comparing E-test and disc diffusion methods with BMD (β = -3.0, 95% CI: -9.2 to 3.2, *p* = 0.35), although this difference did not reach statistical significance, likely due to the small number of included studies. These findings align with multiple comparative studies evaluating VITEK 2 against BMD, demonstrating that VITEK 2 systematically underestimates colistin resistance rates relative to the BMD method [79–81]. These findings have significant clinical implications, given that VITEK 2 is widely utilized in clinical laboratories and healthcare settings for antimicrobial susceptibility testing. The systematic underestimation of colistin resistance by VITEK 2 necessitates caution in interpreting susceptibility results, as misclassification of resistant isolates as susceptible may lead to inappropriate antimicrobial selection. This, in turn, could result in suboptimal treatment outcomes, increased risk of therapeutic failure, and the potential dissemination of resistant pathogens within healthcare environments.

Colistin resistance in Gram-negative bacteria primarily occurs through LPS modification, reducing colistin binding and its bactericidal effect. This is mediated by mutations in PmrAB and PhoPQ two-component systems or plasmid-borne *mcr* genes [11]. Our analysis identified 20 studies reporting on the prevalence of *mcr*-mediated plasmid resistance among colistin-resistant isolates (Table 3). Among these, *mcr-1* remains the most frequently detected resistance gene, particularly in clinically relevant *E. coli*, whereas *mcr-2* appeared only sporadically. The horizontal transfer of *mcr* genes is particularly concerning, as it facilitates the rapid and widespread dissemination of colistin resistance, even in the absence of colistin exposure, across diverse bacterial species. This is consistent with a recent systematic review on *mcr* gene dissemination in Arab countries [82], which demonstrated that *E. coli* is the most common Gram-negative species harboring *mcr* genes in clinical specimens, followed by *K. pneumoniae*. It also highlights that among the various mcr gene variants, *mcr-1* remains the most prevalent and widely distributed across bacterial species and geographic regions.

The horizontal dissemination of *mcr* genes occurs predominantly through two well-characterized mechanisms: (1) the spread of conserved plasmid backbones across genetically diverse bacterial strains, and (2) the mobilization of genetic elements—such as insertion sequences and transposons—that enable the integration and transfer of mcr genes between distinct plasmid types [83]. Among the studies included in this review, only one focused on the genomic characterization of colistin-resistant isolates and demonstrated the presence of *mcr-1* on a conjugative IncHI2/IncHI2A plasmid—a plasmid family known for facilitating inter-strain transfer of resistance genes [70]. This finding underscores the need for further molecular epidemiological investigations to map the dissemination dynamics of *mcr* in Egypt. A deeper understanding of horizontal gene transfer mechanisms is essential, as it elucidates the pathways through which resistance genes spread, informs surveillance and containment strategies, and supports the broader One Health framework for addressing antimicrobial resistance.

The growing concern over the high and increasing rate of colistin resistance in Egypt underscores the urgent need for robust antimicrobial stewardship and effective infection control measures. In parallel, there is a pressing demand to explore alternative strategies to combat multidrug-resistant pathogens. Among these, phage therapy and CRISPR-Cas systems have shown significant promise. Notably, phage therapy has demonstrated encouraging potential in targeting and eliminating *mcr*-harboring colistin-resistant isolates [84, 85], offering a viable alternative where traditional antibiotics fail. Similarly, CRISPR-Cas systems provide a powerful and precise platform to counter antimicrobial resistance by selectively eliminating resistance genes or plasmids. Intriguingly, these systems can naturally occur on mobile genetic elements (MGEs) [86, 87]. Engineered CRISPR-Cas components can be delivered via MGEs, enabling horizontal transfer between bacteria—similar to how resistance genes spread—and thereby enhancing their potential for broad-scale application.

### Study limitations

This meta-analysis has some limitations. First, colistin resistance could not be stratified by species due to the limited number of studies that employed the BMD method for colistin testing in both total recovered and carbapenem-resistant isolates. Second, the multivariate meta-regression model accounted for 53% of the heterogeneity (R² = 0.53). The remaining heterogeneity may be attributed to differences in clinical settings (e.g., ICU vs. general wards), patterns of colistin use, and variations in antimicrobial stewardship and infection control practices across hospitals. Third, while we systematically reviewed all available Egyptian studies investigating *mcr* gene variants among clinically isolated Gram-negative bacteria, the majority of these studies used conventional PCR with primers specifically designed to detect *mcr-1* and *mcr-2*, with a few exceptions. One study employed primers targeting *mcr-1* through *mcr-5* [44], and another utilized whole-genome sequencing [70]. However, neither of these studies identified *mcr* variants beyond *mcr-1* and *mcr-2*. Given these limited data on additional *mcr* variants, further investigation is warranted.

## Conclusion

Colistin resistance was detected in 9% of recovered Gram-negative bacteria in Egypt, with a significant increase to 31% among carbapenem-resistant isolates, as determined by BMD. This rising trend highlights the strong selective pressure imposed by frequent colistin use in carbapenem-resistant infections, fostering the emergence of resistant strains. Over time, the increasing reliance on colistin therapy, along with the spread of plasmid-mediated resistance, has contributed to this escalation. Notably, the *mcr-1* gene was identified as the predominant plasmid-mediated colistin resistance determinant, particularly in *E. coli*, while *mcr-2* remained rare. These findings emphasize the urgent need for robust antimicrobial stewardship programs and strengthened infection control measures, particularly in underserved regions. They also highlight the importance of investing in novel or adjunctive therapies—such as phage therapy, and CRISPR-Cas-based approaches—to preserve the efficacy of last-resort antibiotics like colistin.

## Electronic supplementary material

Below is the link to the electronic supplementary material.

Supplementary Material 1

## Data Availability

All data generated and analyzed throughout this study were included either in this article or its supplementary information file.

## Abbreviations

GNB: Gram-negative bacteria
MDR: Multidrug-resistant
LPS: Lipopolysaccharide
pEtN: Phosphoethanolamine
L-Ara4N: 4-Amino-4-deoxy-L-arabinose
BMD: Broth microdilution
CLSI: Clinical and Laboratory Standards Institute
EUCAST: European Committee on Antimicrobial Susceptibility Testing
PRISMA: Preferred Reporting Items for Systematic Reviews and Meta-Analyses
XDR: Extensively drug-resistant
