# Supplementary material for: Prevalence, trends, and molecular insights into colistin resistance among gram-negative bacteria in Egypt: a systematic review and meta-analysis
Source: Ann Clin Microbiol Antimicrob. 2025 May 10;24:32. doi: 10.1186/s12941-025-00799-3 (PMC12065219; doi:10.1186/s12941-025-00799-3)
Supplement: Supplementary file 1 — Supplementary Material 1 [file 12941_2025_799_MOESM1_ESM.docx]

**Additional file**

**Prevalence, Trends, and Molecular Insights into Colistin Resistance Among Gram-Negative Bacteria in Egypt: A Systematic Review and Meta-Analysis**

**Table S1: The detailed search strategy**

| **Category** | **Search Terms** |
| --- | --- |
| **Colistin Terms** | Colistin OR Polymyxin OR “Polymyxin E” |
| **Gram negative Terms** | "Gram-negative" OR "Gram negative" OR Enterobacterales OR Enterobacteriaceae OR "Escherichia coli" OR "E. coli" OR Klebsiella OR "Klebsiella pneumoniae" OR Enterobacter OR Serratia OR Proteus OR Morganella OR Providencia OR Pseudomonas OR Acinetobacter |
| **Egypt term** | Egypt* |

**Table S2: The Detailed search strategy for Scopus and PubMed Databases**

| **Database** | **Search Strategy** |
| --- | --- |
| **Scopus** | TITLE-ABS-KEY (Colistin OR Polymyxin OR "Polymyxin E") AND TITLE-ABS-KEY ("Gram-negative" OR "Gram negative" OR Enterobacterales OR Enterobacteriaceae OR "Escherichia coli" OR "E. coli" OR Klebsiella OR "Klebsiella pneumoniae" OR Enterobacter OR Serratia OR Proteus OR Morganella OR Providencia OR Pseudomonas OR Acinetobacter) AND TITLE-ABS-KEY (Egypt*) AND (PUBYEAR > 2013 AND PUBYEAR < 2025) |
| **PubMed** | ((Colistin[Title/Abstract] OR Polymyxin[Title/Abstract] OR "Polymyxin E"[Title/Abstract]) AND ("Gram-negative"[Title/Abstract] OR "Gram negative"[Title/Abstract] OR Enterobacterales[Title/Abstract] OR Enterobacteriaceae[Title/Abstract] OR "Escherichia coli"[Title/Abstract] OR "E. coli"[Title/Abstract] OR Klebsiella[Title/Abstract] OR "Klebsiella pneumoniae"[Title/Abstract] OR Enterobacter[Title/Abstract] OR Serratia[Title/Abstract] OR Proteus[Title/Abstract] OR Morganella[Title/Abstract] OR Providencia[Title/Abstract] OR Pseudomonas[Title/Abstract] OR Acinetobacter[Title/Abstract]))  AND (Egypt[Title/Abstract]) AND ("2014/01/01"[Date - Publication] : "2024/12/05"[Date - Publication]) |

**Table S3: The 27-Item Checklist of the PRISMA Statement**

| Section/topic | Item No | Checklist item | Reported on page No |
| --- | --- | --- | --- |
| Title | | | |
| Title | 1 | Identify the report as a systematic review, meta-analysis, or both | 1 |
| Abstract | | | |
| Structured summary | 2 | Provide a structured summary including, as applicable, background, objectives, data sources, study eligibility criteria, participants, interventions, study appraisal and synthesis methods, results, limitations, conclusions and implications of key findings, systematic review registration number | 2,3 |
| Introduction | | | |
| Rationale | 3 | Describe the rationale for the review in the context of what is already known | 5 |
| Objectives | 4 | Provide an explicit statement of questions being addressed with reference to participants, interventions, comparisons, outcomes, and study design (PICOS) | 5 |
| Methods | | | |
| Protocol and registration | 5 | Indicate if a review protocol exists, if and where it can be accessed (such as web address), and, if available, provide registration information including registration number. | - |
| Eligibility criteria | 6 | Specify study characteristics (such as PICOS, length of follow-up) and report characteristics (such as years considered, language, publication status) used as criteria for eligibility, giving rationale | 6 |
| Information sources | 7 | Describe all information sources (such as databases with dates of coverage, contact with study authors to identify additional studies) in the search and date last searched | 5 |
| Search | 8 | Present full electronic search strategy for at least one database, including any limits used, such that it could be repeated | Tables S1 and S2 |
| Study selection | 9 | State the process for selecting studies (that is, screening, eligibility, included in systematic review, and, if applicable, included in the meta-analysis) | 7 |
| Data collection process | 10 | Describe method of data extraction from reports (such as piloted forms, independently, in duplicate) and any processes for obtaining and confirming data from investigators | 7 |
| Data items | 11 | List and define all variables for which data were sought (such as PICOS, funding sources) and any assumptions and simplifications made | 7 |
| Risk of bias in individual studies | 12 | Describe methods used for assessing risk of bias of individual studies (including specification of whether this was done at the study or outcome level), and how this information is to be used in any data synthesis | 7  **Table S4.** |
| Summary measures | 13 | State the principal summary measures (such as risk ratio, difference in means). | 8 |
| Synthesis of results | 14 | Describe the methods of handling data and combining results of studies, if done, including measures of consistency (such as I^2^ statistic) for each meta-analysis | 8 |
| Risk of bias across studies | 15 | Specify any assessment of risk of bias that may affect the cumulative evidence (such as publication bias, selective reporting within studies) | - |
| Additional analyses | 16 | Describe methods of additional analyses (such as sensitivity or subgroup analyses, meta-regression), if done, indicating which were pre-specified | 7 |
| Results | | | |
| Study selection | 17 | Give numbers of studies screened, assessed for eligibility, and included in the review, with reasons for exclusions at each stage, ideally with a flow diagram | 8&9 &  Fig. 1 |
| Study characteristics | 18 | For each study, present characteristics for which data were extracted (such as study size, PICOS, follow-up period) and provide the citations | 9 and  Table 1. |
| Risk of bias within studies | 19 | Present data on risk of bias of each study and, if available, any outcome-level assessment (see item 12). | Table 1. |
| Results of individual studies | 20 | For all outcomes considered (benefits or harms), present for each study (a) simple summary data for each intervention group and (b) effect estimates and confidence intervals, ideally with a forest plot | Tables 2-3 and Figs 2and 3 |
| Synthesis of results | 21 | Present results of each meta-analysis done, including confidence intervals and measures of consistency | Tables 2-3 and Figs 2and 3 |
| Risk of bias across studies | 22 | Present results of any assessment of risk of bias across studies (see item 15) | - |
| Additional analysis | 23 | Give results of additional analyses, if done (such as sensitivity or subgroup analyses, meta-regression) (see item 16) | 17 &18 &Table 2 |
| Discussion | | | |
| Summary of evidence | 24 | Summarize the main findings including the strength of evidence for each main outcome; consider their relevance to key groups (such as health care providers, users, and policy makers) | 22 |
| Limitations | 25 | Discuss limitations at study and outcome level (such as risk of bias), and at review level (such as incomplete retrieval of identified research, reporting bias) | 25 |
| Conclusions | 26 | Provide a general interpretation of the results in the context of other evidence, and implications for future research | 26 |
| Funding | | | |
| Funding | 27 | Describe sources of funding for the systematic review and other support (such as supply of data) and role of funders for the systematic review | 27 |

**Table S4**: The checklist items for Joanna Briggs's critical appraisal tool for prevalence studies

|  | Yes | No | Unclear | Not applicable |
| --- | --- | --- | --- | --- |
| 1. Was the sample frame appropriate to address the target population? | □ | □ | □ | □ |
| 1. Were study participants sampled in an appropriate way? | □ | □ | □ | □ |
| 1. Was the sample size adequate? | □ | □ | □ | □ |
| 1. Were the study subjects and the setting described in detail? | □ | □ | □ | □ |
| 1. Was the data analysis conducted with sufficient coverage of the identified sample? | □ | □ | □ | □ |
| 1. Were valid methods used for the identification of the condition? | □ | □ | □ | □ |
| 1. Was the condition measured in a standard, reliable way for all participants? | □ | □ | □ | □ |
| 1. Was there appropriate statistical analysis? | □ | □ | □ | □ |
| 1. Was the response rate adequate, and if not, was the low response rate managed appropriately? | □ | □ | □ | □ |

**Table S5**: Detailed quality scores of the included studies based on the Joanna Briggs Institute Critical Appraisal Tool for Prevalence Studies.

| **Last name of first author** | **Q1** | **Q2** | **Q3** | **Q4** | **Q5** | **Q6** | **Q7** | **Q8** | **Quality score (Out of 8) *** |
| --- | --- | --- | --- | --- | --- | --- | --- | --- | --- |
| **Shawky [1]** | Y | Y | N | N | Y | N | Y | Y | 5 |
| **Azzab [2]** | Y | Y | N | Y | Y | N | Y | Y | 6 |
| **Alkasaby [3]** | Y | Y | Y | Y | Y | N | Y | Y | 7 |
| **Assem [4]** | Y | Y | N | Y | Y | N | Y | Y | 6 |
| **Ghonaim [5]** | Y | Y | N | Y | Y | N | Y | Y | *6* |
| **Abdulall [6]** | Y | Y | N | Y | Y | N | Y | Y | *6* |
| **Abdulzahra [7]** | Y | Y | N | Y | Y |  | Y | Y | *7* |
| **El-Mahallawy[8]** | Y | Y | N | Y | Y | N | Y | Y | *6* |
| **El-Masry[9]** | Y | Y | N | Y | Y | N | Y | Y | *6* |
| **Awad[10]** | Y | Y | N | Y | Y | N | Y | Y | *6* |
| **Emara[11]** | Y | Y | Y | Y | Y | N | Y | Y | *7* |
| **Mokhtar [12]** | Y | Y | Y | Y | Y | Y | Y | Y | *8* |
| **Sokkary[13]** | Y | Y | Y | Y | Y | Y | Y | Y | *8* |
| **Zafer [14]** | Y | Y | Y | Y | Y | Y | Y | Y | *8* |
| **Basha[15]** | Y | Y | Y | Y | Y | Y | Y | Y | *8* |
| **El-Baky [16]** | Y | Y | N | Y | Y | N | Y | Y | *6* |
| **Fam [17]** | Y | Y | N | Y | Y | Y | Y | Y | *7* |
| **Rabie [18]** | Y | Y | Y | Y | Y | Y | Y | Y | *8* |
| **Raheel [19]** | Y | Y | N | Y | Y | Y | Y | Y | *7* |
| **Shabban [20]** | Y | Y | N | Y | Y | N | Y | Y | *6* |
| **El-Mokhtar [21]** | Y | Y | Y | Y | Y | Y | Y | Y | *8* |
| **Elshimy [22]** | Y | Y | Y | Y | Y | N | Y | Y | *7* |
| **Ibrahim[23]** | Y | Y | Y | Y | Y | Y | Y | Y | *8* |
| **Khattab [24]** | Y | Y | Y | Y | Y | Y | Y | Y | *8* |
| **Mashalya [25]** | Y | Y | Y | Y | Y | Y | Y | Y | *8* |
| **Negm [26]** | Y | Y | Y | Y | Y | N | Y | Y | *7* |
| **Ajlan [27]** | Y | Y | Y | Y | Y |  | Y | Y | *8* |
| **Badran [28]** | Y | Y | N | Y | Y | N | Y | Y | *6* |
| **Defrawy [29]** | Y | Y | N | Y | Y | N | Y | Y | *6* |
| **El-Din [30]** | Y | Y | N | Y | Y | N | Y | Y | *6* |
| **Gaballah [31]** | Y | Y | Y | Y | Y | N | Y | Y | *7* |
| **El-Mahallawy [32]** | Y | Y | Y | Y | Y | Y | Y | Y | *8* |
| **Mohamed [33]** | Y | Y | N | Y | Y | N | Y | Y | *5* |
| **Mostafa [34]** | Y | Y | Y | Y | Y | N | Y | Y | *7* |
| **Ramadan [35]** | Y | Y | N | Y | Y | N | Y | Y | *6* |
| **Shrief [36]** | Y | Y | N | Y | Y | Y | Y | Y | *7* |
| **Sorour [37]** | Y | Y | Y | Y | Y | Y | Y | Y | *8* |
| **Abdelbary [38]** | Y | Y | N | Y | Y | Y | Y | Y | *7* |
| **Abozahra [39]** | Y | Y | N | Y | Y | V | Y | Y | *7* |
| **Elshamy [40]** | Y | Y | N | Y | Y | Y | Y | Y | *7* |
| **Khatib [41]** | Y | Y | Y | Y | Y | Y | Y | Y | *8* |
| **Mahmoud[42]** | Y | Y | N | Y | Y | Y | Y | Y | 7 |
| **Mohamed [43]** | Y | Y | Y | Y | Y | N | Y | Y | *7* |
| **Abdel-Aty [44]** | Y | Y | Y | Y | Y | Y | Y | Y | *8* |
| **Afify [45]** | Y | Y | Y | Y | Y | Y | Y | Y | *8* |
| **Ali[46]** | Y | Y | N | Y | Y | N | Y | Y | *6* |
| **Alshaikh [47]** | Y | Y | Y | Y | Y | Y | Y | Y | *8* |
| **Edward [48]** | Y | Y | Y | Y | Y | Y | Y | Y | *8* |
| **El-Kholy [49]** | Y | Y | N | Y | Y | Y | Y | Y | *7* |
| **Makled[50]** | Y | Y | N | Y | Y | N | Y | Y | *6* |
| **Mohamed[51]** | Y | Y | N | Y | Y | N | Y | Y | *6* |
| **Elnahriry[52]** | Y | Y | Y | Y | Y | Y | Y | Y | *8* |
| **Attalla, [53]** | Y | Y | Y | Y | Y | Y | Y | Y | *8* |
| **Osama [54]** | Y | Y | N | Y | Y | N | Y | Y | *6* |
| **Al-Agamy [55]** | Y | Y | N | Y | Y | N | Y | Y | *6* |
| Y “YES”, N “No” * Question 9 of the original JBI checklist was omitted as it was not applicable. | | | | | | | | | |

1. Shawky SM, Abdallah A, Khouly M. Antimicrobial activity of Colistin and Tiegecycline against carbapenem-resistant Klebsiella pneumoniae clinical isolates in Alexandria, Egypt. IntJCurrMicrobiolAppSci. 2015;4:731–42.

2. Azzab MM, El-Sokkary RH, Tawfeek MM, Gebriel MG. Multidrug-resistant bacteria among patients with ventilatorassociated pneumonia in an emergency intensive care unit, Egypt. East Mediterr Health J. 2017;22:894–903.

3. Alkasaby NM, El Sayed Zaki M. Molecular Study of Acinetobacter baumannii Isolates for Metallo- β-Lactamases and Extended-Spectrum- β-Lactamases Genes in Intensive Care Unit, Mansoura University Hospital, Egypt. Int J Microbiol. 2017;2017.

4. Assem M, Abdalla WM-N, Elsherif R, Saad A, Kadry ID, Hasanin A, et al. Emergence of Gram-Negative Bacilli with Concomitant blaNDM-1- and blaOXA-48-Like Genes in Egypt. Am J Intern Med. 2017;5:1.

5. Ghonaim A, Elgohary T. The Rate of Extended-Spectrum B-Lactamases among Commonly Isolated Enterobacteriaceae in Cancer Patients in Zagazig University Hospitals. J Microbiol Res. 2017;7:31–8.

6. Abdulall AK, Tawfick MM, El Manakhly AR, El Kholy A. Carbapenem-resistant Gram-negative bacteria associated with catheter-related bloodstream infections in three intensive care units in Egypt. Eur J Clin Microbiol Infect Dis. 2018;37:1647–52.

7. Abdulzahra AT, Khalil MAF, Elkhatib WF. First report of colistin resistance among carbapenem-resistant Acinetobacter baumannii isolates recovered from hospitalized patients in Egypt. New Microbes New Infect. 2018;26:53.

8. ElMahallawy HA, Zafer MM, Amin MA, Ragab MM, Al-Agamy MH. Spread of carbapenem resistant Enterobacteriaceae at tertiary care cancer hospital in Egypt. Infect Dis (London, England). 2018;50:560–4.

9. El-Masry EA, El-Masry HA, El-Masry E. Characterization of Carbapenem-resistant Acinetobacter baumannii Isolated from Intensive Care Unit, Egypt. Egypt J Med Microbiol. 2018;27:85–91.

10. Awad S, Ghanem S, Helal M, Elgedawy G, Khalil F, Fikry M, et al. Phenotypic and genotypic characteristics of communityacquired and hospital-acquired carbapenem-resistant Enterobacteriaceae in patients with liver cirrhosis at the National Liver Institute of Egypt. Can J Infect Control. 2019;:100–3.

11. Emara MMM, Abd-Elmonsef MME, Abo Elnasr LM, Elfeky AAEE. Study of mcr-1 Gene-Mediated Colistin-Resistance in Gram-Negative Isolates in Egypt. Egypt J Med Microbiol. 2019;28:9–16.

12. El-Mokhtar MA, Mandour SA, Shahat AA. Colistin resistance among multidrug-resistant E. coli isolated from Upper Egypt. Egypt J Med Microbiol. 2019;28:11–7.

13. El-Sokkary RH, Gebriel MG. Colistin Susceptibility and the Effect of Colistin-sulfadiazine Combination among Multidrug Resistant E. coli and K. pneumoniae at Egyptian Intensive Care Units. Egypt J Med Microbiol. 2019;28:87–93.

14. Zafer MM, El-Mahallawy HA, Abdulhak A, Amin MA, Al-Agamy MH, Radwan HH. Emergence of colistin resistance in multidrug-resistant Klebsiella pneumoniae and Escherichia coli strains isolated from cancer patients. Ann Clin Microbiol Antimicrob. 2019;18:1–8.

15. Basha AM, El-Sherbiny GM, Mabrouk MI. Phenotypic characterization of the Egyptian isolates “extensively drug-resistant Pseudomonas aeruginosa” and detection of their metallo-β-lactamases encoding genes. Bull Natl Res Cent. 2020;44:1–11.

16. Abd El-Baky RM, Masoud SM, Mohamed DS, Waly NGFM, Shafik EA, Mohareb DA, et al. Prevalence and Some Possible Mechanisms of Colistin Resistance Among Multidrug-Resistant and Extensively Drug-Resistant Pseudomonas aeruginosa. Infect Drug Resist. 2020;13:323–32.

17. Fam NS, Gamal D, Mohamed SH, Wasfy RM, Soliman MS, El-Kholy AA, et al. Molecular Characterization of Carbapenem/Colistin-Resistant Acinetobacter baumannii Clinical Isolates from Egypt by Whole-Genome Sequencing. Infect Drug Resist. 2020;13:4487–93.

18. Rabie RA, Abdallah AL. Plasmid mediated colistin resistant genes mcr-1 and mcr-2 among Escherichia coli and Klebsiella Pneumoniae isolates at Zagazig University Hospitals, Egypt. Egypt J Med Microbiol. 2020;29:61–6.

19. Raheel AS, Mohamed HA, Hessam WF, Abbadi SH, El Sayed AE. Detection of carbapenemase enzymes and genes among carbapenem-resistant Enterobacteriaceae isolates in Suez Canal University Hospitals in Ismailia ,Egypt. Microbes Infect Dis. 2020;1:24–33.

20. Shabban M, Fahim NAE, Montasser K, Abo El Magd NM. Resistance to colistin mediated by mcr-1 among multidrug resistant gram negative pathogens at a tertiary care hospital, Egypt. J Pure Appl Microbiol. 2020;14:1125–32.

21. El-Mokhtar MA, Daef E, Hussein AARM, Hashem MK, Hassan HM. Emergence of Nosocomial Pneumonia Caused by Colistin-Resistant Escherichia coli in Patients Admitted to Chest Intensive Care Unit. Antibiotics. 2021;10:226.

22. Elshimy R, Zedan H, Elmorsy TH, Khattab RA. A Study on Multidrug-Resistant Escherichia coli Clinical Isolates from Different Hospitals in Greater Cairo. Microb Drug Resist. 2021;27:1420–32.

23. Ibrahim ER, Ahmed YM, Mohamed AK, Ibrahim WAEL. Detection of colistin resistant Gram negative bacilli in intensive care unit patients admitted to Ain Shams University Hospitals. Microbes Infect Dis. 2021;2:92–9.

24. Khattab SM, El-Sweify MA, Metwally LA, El-Azab SZ, Hashem AAE. Detection of plasmid-mediated colistin resistance in carbapenem-resistant Escherichia coli and Klebsiella pneumoniae isolates in Suez Canal University Hospitals. Microbes Infect Dis. 2021;2:497–507.

25. Mashaly GES, Mashaly MES. Colistin-heteroresistance in carbapenemase-producing Enterobacter species causing hospital-acquired infections among Egyptian patients. J Glob Antimicrob Resist. 2021;24:108–13.

26. Negm EM, Mowafy SMS, Mohammed AA, Amer MG, Tawfik AE, Ibrahim AES, et al. Antibiograms of intensive care units at an Egyptian tertiary care hospital. Egypt J Bronchol. 2021;15:15.

27. Ajlan SE, Elmahdy EE, Sleem AS. Assessment of Colistin Susceptibility among Carbapenem-Resistant Clinical Isolates. Egypt J Med Microbiol. 2022;31:109–16.

28. Badran SG, Malek MM, Ateya RM, Afifi AH, Magdy MM, Elgharabawy ES. Susceptibility of carbapenem-resistant Enterobacterales isolates to new antibiotics from a tertiary care hospital, Egypt: A matter of hope. J Infect Dev Ctries. 2022;16:1852–9.

29. El-Defrawy I, Aitta AA, Fam N, Khaled M, Madany N, Damarawy M El, et al. In Vitro Activity of Single and Combined Antibiotics against Carbapenem Resistant Enterobacteriaceae Clinical Isolates in Relation to their Resistance Genes. Open Access Maced J Med Sci. 2022;10 A:1600–7.

30. El-Din AN, Anwar S, Esmat MM. Emergence of Colistin-resistant Pseudomonas aeruginasa in Sohag University Hospitals, Egypt. Microbes Infect Dis. 2022;3:958–71.

31. Gaballah AH, Shawky S, Amer AN. Microbiological profiles of neonatal sepsis in northern Egypt. Microbes Infect Dis. 2022;3:645–56.

32. El-Mahallawy HA, El Swify M, Abdul Hak A, Zafer MM. Increasing trends of colistin resistance in patients at high-risk of carbapenem-resistant Enterobacteriaceae. Ann Med. 2022;54:1.

33. Mohamed NMK, El-Baghdady KZ, El-Kholy E, Fahmy GM. Prevalence of carbapenemase genes in extreme drug resistant Pseudomonas aeruginosa isolated from ICU in Egypt. African J Biol Sci. 2022;18:39–51.

34. Mostafa SH, Saleh SE, Hamed SM, Aboshanab KM. Febrile illness of bacterial etiology in a public fever hospital in Egypt: High burden of multidrug resistance and WHO priority Gram negative pathogens. Germs. 2022;12:75–85.

35. Ramadan RA, Bedawy AM, Negm EM, Hassan TH, Ibrahim DA, Elsheikh SM, et al. Carbapenem-Resistant Klebsiella pneumoniae Among Patients with Ventilator-Associated Pneumonia: Evaluation of Antibiotic Combinations and Susceptibility to New Antibiotics. Infect Drug Resist. 2022;15:3537–48.

36. Shrief R, El-Ashry AH, Mahmoud R, El-Mahdy R. Effect of Colistin, Fosfomycin and Meropenem/Vaborbactam on Carbapenem-Resistant Enterobacterales in Egypt: A Cross-Sectional Study. Infect Drug Resist. 2022;15:6203.

37. Sorour AE, Ibrahim KAA, Hegab AS. Prevalence of Acquired Colistin Resistance among Gram Negative Bacilli Isolated from Patients Admitted at Cairo University Hospitals. Egypt J Med Microbiol. 2022;31:97–104.

38. Abdelbary ER, Elsaghier AM, El-Baky RMA, Waly NGFM, Ramadan M, Abd-Elsamea FS, et al. First Emergence of NDM-5 and OqxAB Efflux Pumps Among Multidrug-Resistant Klebsiella pneumoniae Isolated from Pediatric Patients in Assiut, Egypt. Infect Drug Resist. 2023;16:5965–76.

39. Abozahra R, Gaballah A, Abdelhamid SM. Prevalence of the colistin resistance gene MCR-1 in colistin-resistant Klebsiella pneumoniae in Egypt. AIMS Microbiol. 2023;9:177–94.

40. Elshamy AA, Saleh SE, Aboshanab KM, Aboulwafa MM, Hassouna NA. In Vitro Meropenem/Antibiotic and Meropenem/Bacteriophage Combinations Against Carbapenem-Resistant Gram-Negative Uropathogens. Arch Pharm Sci Ain Shams Univ. 2023;7:19–30.

41. Mohamed El-Khatib A, Farouk Basyony A, Ali El-Gharib K. Detection of mcr-1 to mcr-5 Genes-Mediated Colistin-Resistance in Gram-Negative Clinical Isolates. Al-Azhar Med J. 2023;52:943–56.

42. Mahmoud FM, Moustafa NM, Gaber SA, Elsaid RG, Mohamed RA. Molecular mechanisms of colistin resistance among multi-drug resistant (MDR) Klebsiella pneumoniae and Escherichia coli isolated from ICU patients and their susceptibility towards eravacycline. Microbes Infect Dis. 2023;4:127–37.

43. Mohamed N, Ghazal A, Ahmed AAH, Zaki A. Prevalence and determinants of antimicrobial resistance of pathogens isolated from cancer patients in an intensive care unit in Alexandria, Egypt. J Egypt Public Health Assoc. 2023;98:9.

44. Abdel-Aty HF, El-Batal HM, Gohar NM. Assessment of colistin resistance among nosocomial multidrug-resistant Gram-negative bacilli isolated from different clinical samples. Microbes Infect Dis. 2024;5:1494–505.

45. Afify FA, Shata AH, Aboelnaga N, Osama D, Elsayed SW, Saif NA, et al. Emergence of carbapenem resistant gram-negative pathogens with high rate of colistin resistance in Egypt: A cross sectional study to assess resistance trends during the COVID-19 pandemic. J Genet Eng Biotechnol. 2024;22:100351.

46. Ali SESI, Mahmoud NAS, Elamin MM, Amr GES, Mahrous HKA. Assessment of carbapenem resistant Klebsiella pneumoniae in intensive care unit of Zagazig University Hospitals. Microbes Infect Dis. 2024;5:282–94.

47. Alshaikh SA, El-banna T, Sonbol F, Farghali MH. Correlation between antimicrobial resistance, biofilm formation, and virulence determinants in uropathogenic Escherichia coli from Egyptian hospital. Ann Clin Microbiol Antimicrob. 2024;23.

48. Edward EA, El Shehawy MR, Abouelfetouh A, Aboulmagd E. Phenotypic and molecular characterization of extended spectrum- and metallo- beta lactamase producing Pseudomonas aeruginosa clinical isolates from Egypt. Infection. 2024;52:2399–414.

49. El-Kholy AT, El-Kholy MA, Omar H, Aboulmagd E. Co-existence of antibiotic resistance and virulence factors in carbapenem resistant Klebsiella pneumoniae clinical isolates from Alexandria, Egypt. BMC Microbiol. 2024;24:466.

50. Makled AF, Younes HEB, Ghonaim MM, Shaalan AK, El-Mahdy EES. Colistin and carbapenem resistance among Pseudomonas and Acinetobacter clinical isolates in Menoufia University Hospitals. Microbes Infect Dis. 2024;5:301–13.

51. Mohamed TA, Afifi S, Hassaneen A, Radwan M, Ateya RM. Carbapenem-resistant Klebsiella pneumoniae in COVID-19 patients admitted to intensive care units of Zagazig University Hospitals. Microbes Infect Dis. 2024;0:0–0.

52. Elnahriry SS, Khalifa HO, Soliman AM, Ahmed AM, Hussein AM, Shimamoto T, et al. Emergence of Plasmid-Mediated Colistin Resistance Gene mcr-1 in a Clinical Escherichia coli Isolate from Egypt. Antimicrob Agents Chemother. 2016;60:3249–50.

53. Attalla ET, Khalil AM, Zakaria AS, Baker DJ, Mohamed NM. Genomic characterization of colistin-resistant Klebsiella pneumoniae isolated from intensive care unit patients in Egypt. Ann Clin Microbiol Antimicrob. 2023;22.

54. Osama R, Bakeer W, Fadel S, Amin M. Association of carbapenem and colistin resistance in pathogenic Gram negative bacteria. J Pure Appl Microbiol. 2019;13:733–9.

55. Al-Agamy MH, Khalaf NG, Tawfick MM, Shibl AM, El Kholy AA. Molecular characterization of carbapenem-insensitive Acinetobacter baumannii in Egypt. Int J Infect Dis. 2014;22:49–54.
